# NADPH Oxidase and Guanylate Binding Protein 5 Restrict Survival of Avirulent Type III Strains of Toxoplasma gondii in Naive Macrophages

**DOI:** 10.1128/mBio.01393-18

**Published:** 2018-08-28

**Authors:** Sumit K. Matta, Kelley Patten, Quiling Wang, Bae-Hoon Kim, John D. MacMicking, L. David Sibley

**Affiliations:** a Department of Molecular Microbiology, Washington University School of Medicine in St. Louis, St. Louis, Missouri, USA; b Howard Hughes Medical Institute, West Haven, Connecticut, USA; c Yale Systems Biology Institute, West Haven, Connecticut, USA; d Department of Immunobiology, Yale University School of Medicine, West Haven, Connecticut, USA; e Department of Microbial Pathogenesis, Yale University School of Medicine, West Haven, Connecticut, USA; Albert Einstein College of Medicine

**Keywords:** autophagy, chronic granulomatous disease, innate immunity, interferons, intracellular parasites, membrane damage, reactive oxygen species, toxoplasmosis

## Abstract

Phagocytic cells are the first line of innate defense against intracellular pathogens, and yet Toxoplasma gondii is renowned for its ability to survive in macrophages, although this paradigm is based on virulent type I parasites. Surprisingly, we find that avirulent type III parasites are preferentially cleared in naive macrophages, independent of gamma interferon (IFN-γ) activation. The ability of naive macrophages to clear type III parasites was dependent on enhanced activity of NADPH oxidase (Nox)-generated reactive oxygen species (ROS) and induction of guanylate binding protein 5 (Gbp5). Macrophages infected with type III parasites (CTG strain) showed a time-dependent increase in intracellular ROS generation that was higher than that induced by type I parasites (GT1 strain). The absence of Nox1 or Nox2, gp91 subunit isoforms of the Nox complex, reversed ROS-mediated clearance of CTG parasites. Consistent with this finding, both Nox1^−/−^ and Nox2^−/−^ mice showed higher susceptibility to CTG infection than wild-type mice. Additionally, Gbp5 expression was induced upon infection and the enhanced clearance of CTG strain parasites was reversed in Gbp5^−/−^ macrophages. Expression of a type I ROP18 allele in CTG prevented clearance in naive macrophages, suggesting that it plays a role counteracting Gbp5. Although ROS and Gbp5 have been linked to activation of the NLRP3 inflammasome, clearance of CTG parasites did not rely on induction of pyroptosis. Collectively, these findings reveal that not all strains of T. gondii are adept at avoiding clearance in macrophages and define new roles for ROS and Gbps in controlling this important intracellular pathogen.

## Abstract (precis)

*Toxoplasma* infections in humans and other mammals are largely controlled by IFN-γ produced by the activated adaptive immune system. However, we still do not completely understand the role of cell-intrinsic functions in controlling *Toxoplasma* or other apicomplexan infections. The present work identifies intrinsic activities in naive macrophages in counteracting T. gondii infection. Using an avirulent strain of T. gondii, we highlight the importance of Nox complexes in conferring protection against parasite infection both *in vitro* and *in vivo*. We also identify Gbp5 as a novel macrophage factor involved in limiting intracellular infection by avirulent strains of T. gondii. The rarity of human infections caused by type III strains suggests that these mechanisms may also be important in controlling human toxoplasmosis. These findings further extend our understanding of host responses and defense mechanisms that act to control parasitic infections at the cellular level.

## INTRODUCTION

Toxoplasma gondii is an obligate intracellular parasite of the apicomplexan phylum, and it is capable of infecting a wide range of mammals, including humans (1). The life cycle includes multiple invasive forms (i.e., tachyzoites, bradyzoites, merozoite, and sporozoites), each of which successfully invades and replicates inside its host cell (2). The parasite avoids phagocytosis and instead actively invades its host cell and thereafter resides in a stable parasitophorous vacuole (PV), where it replicates (3, 4). The PV exhibits several notable features that help explain the survival of T. gondii within hostile cells such as macrophages. First, entry into human macrophages occurs without eliciting a classical respiratory burst (5). Second, the lumen of the vacuole fails to acidify, owing to an absence of delivery of proton pumps to the membrane (6). Third, the vacuole fails to fuse with lysosomes, thus protecting it from their hydrolytic contents (7). The molecular mechanism by which the PV avoids eliciting host cellular responses remains undefined but may stem from active invasion and the formation of the PV membrane by invagination of the plasma membrane (8) and extensive remodeling of its contents to mask identity (9, 10).

In North America and Europe, the population structure of T. gondii is dominated by three clonal lineages known as types I, II, and III (11). Although type I strains are highly studied due to their acute virulence in laboratory mice (12), they are not the major types found in natural infection. Rather, type II strains, which exhibit intermediate levels of virulence in laboratory mice, predominate among animal and human infections in North America and Europe (13–15). Type II strains exhibit intermediate virulence in mice and yet are capable of causing significant disease in humans (13–15). Type III strains are also common in animals, and yet they are extremely rare in humans (13–15), suggesting that they either do not cause infection or at least rarely cause disease. The major differences in mouse virulence among these strain types have been mapped to a polymorphic family of rhoptry kinases including ROP18 and ROP5 (16). These determinants have been shown to play a major role in combating host defense in gamma interferon (IFN-γ)-activated cells, where the acute virulence is attributed to their composite genotypes (17).

IFN-γ is the major resistance determinant that is required to control T. gondii infection in mice (18), and signaling evoked by this cytokine is essential in both hematopoietic and nonhematopoietic cells (19). At the cellular level, IFN-γ exerts its anti-*Toxoplasma* effect by upregulating a variety of interferon-stimulated genes (ISGs), including those for immunity-related GTPases (IRGs), guanylate binding proteins (GBPs), nitric oxide, or autophagy-related clearance, which in turn either control growth or damage the parasite directly (20–23). IFN-γ-induced Irgs disrupt and damage the parasitophorous vacuolar membrane (PVM), leading to parasite killing in mouse macrophages, and this pathway is avoided by virulent type I strains (24, 25). Type I parasites employ ROP18 to phosphorylate Irgs and thus inhibit their recruitment to their vacuole, thereby preventing their damage and clearance in mouse macrophages (26, 27). However, type III parasites lack expression of ROP18 and therefore are rapidly cleared from IFN-γ-activated macrophages, a defect that is restored by complementation with ROP18 (26, 27). Mouse GBPs are also known to target the PVM in IFN-γ-activated cells (28), leading to degradation of the parasite (29). Deletion of the GBP cluster of chromosome 3 makes mice more susceptible to T. gondii infection (30), as does loss of Gbp1 (31) or Gbp2 (32). ROP18 has also been shown to participate in defense against Gbps (31), although the mechanism responsible for this activity is unknown.

Although most Gbps are known to target invading pathogens, Gbp5 can also assemble the NLRP3 inflammasome in the absence of infection, and within infected cells, such assembly could conceivably take place on or near the pathogen vacuole (33). In addition, Gbp2 has been shown to function in activation of the AIM2-dependent inflammasome in response to Francisella novicida (34). Inflammasomes are cytosolic oligomeric protein complexes that often consist of Asc, caspase-1, and an NLR or ALR sensor protein formed in response to cellular danger or pathogen-associated signals. Upon activation, the inflammasome leads to autoproteolysis of procaspase-1 to proteolytically active caspase-1 that in turn cleaves cytosolic prointerleukin-1β (pro-IL-1β) and pro-IL-18 to secretory IL-1β and IL-18, respectively. These cytokines then drive downstream signaling to promote antimicrobial function of macrophages and Th1 adaptive immune responses (35, 36). Inflammasome activation has been implicated in controlling T. gondii infection *in vivo*, where mice lacking NLRP1B (37) or NLRP3 or caspase-1 (38) are more susceptible to type II parasite infection.

Gbp7 has been implicated in the recruitment and assembly of NADPH oxidase complexes to mycobacterium-containing phagosomes (39), suggesting that GBPs may also participate in pathogen control by recruitment of other effector complexes. NADPH oxidases (Nox) are multimeric enzyme complexes that generate superoxide free radicals upon activation (40). Although the Nox complex is expressed in almost every mammalian tissue, its function of producing reactive oxygen species (ROS) in host defense against pathogens is more pronounced in phagocytes, where the Nox2 isoform predominates (41). Upon activation, gp91phox (Nox2) and p22phox subunits that reside in the plasma membrane recruit other cytosolic subunits of the complex (i.e., p40phox, p47phox, and p67phox) to convert NADPH to NADP^+^ and hence generate superoxide radicals (41). When activated, Nox rapidly generates elevated production of ROS (defined as respiratory burst) in response to pattern recognition receptors of invading pathogens (42). ROS production is dependent on expression of Nox1 and Nox2 isoforms in murine bone marrow-derived macrophages (BMDMs) that cross-regulate differentiation profiles of macrophages (43). Mutation in genes involved in NADPH oxidase functioning or assembly causes chronic granulomatous disease (CGD) in humans that typically results in frequent bacterial or fungal infections (44).

Here, we undertook examination of the fate of avirulent type III strains in naive macrophages. We were surprised to find that the paradigm that T. gondii survives in macrophages does not apply uniformly to all strains, but rather, type III strains are highly susceptible to clearance. We demonstrate that in naive macrophages with avirulent type III T. gondii, parasites induced Nox-dependent ROS production and Gbp5 expression. Both of these factors are involved in clearance of type III parasites from naive macrophages independent of prior activation with IFN-γ and independent of classical inflammasome activation that can be primed by LPS (33). These findings reveal novel roles for cell-intrinsic factors in controlling intracellular pathogens and suggest that virulent strains of T. gondii have additional effectors that block these mechanisms.

## RESULTS

### Clearance of CTG (type III) parasites in naive macrophages.

**FIG 1:**
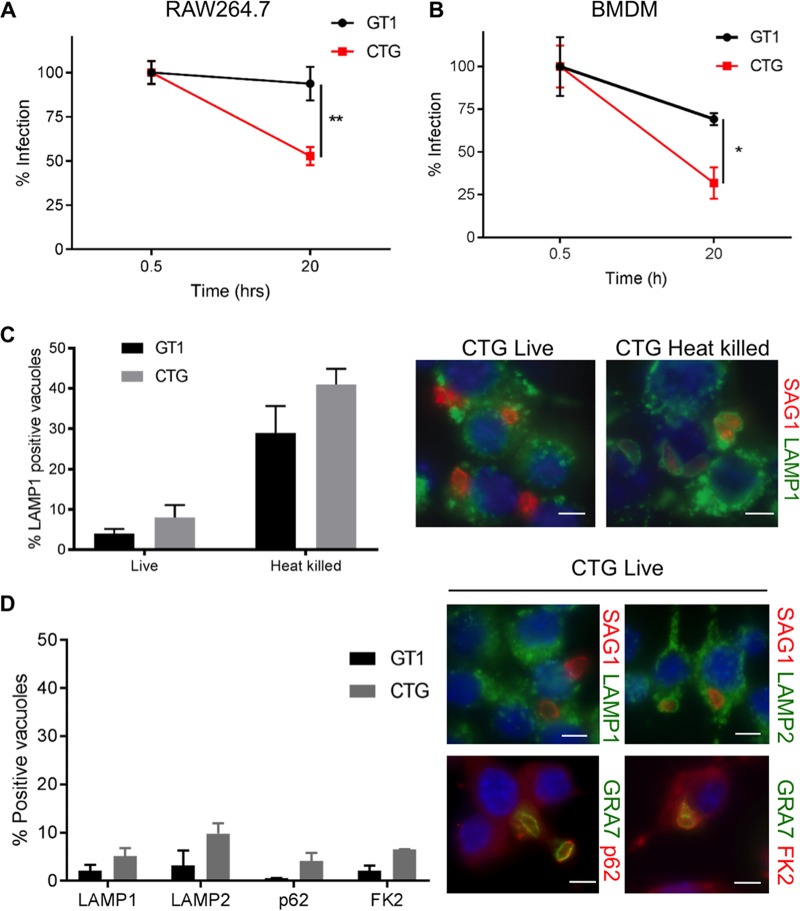
Survival of T. gondii in naive macrophages. (A) Intracellular survival of GT1 (type I) and CTG (type III) parasites in naive RAW 264.7 cells at 20 h expressed as percentage of initial infection at 0.5 h. (B) Intracellular survival of GT1 (type I) and CTG (type III) parasites in naive bone marrow-derived macrophages (BMDMs) at 20 h expressed as percentage of initial infection at 0.5 h. Macrophages were challenged with parasites at an MOI of 0.5, washed, fixed, and stained with SAG1 to detect parasites and LAMP1 to detect host cell lysosomes (used for identifying host cells in high-content image analysis). Values represent means ± standard errors of the means from three independent replicates with at least 50 fields per replicate. By unpaired Student’s *t* test, * and ** represent significant differences at *P* < 0.05 and *P* < 0.01, respectively. (C) Percentage of LAMP1-positive live or heat-killed GT1 or CTG parasites in BMDMs at 2 h postinfection. Values represent means ± standard errors of the means from three independent replicates. Representative images to the right showing parasites stained with SAG1 (MAb DG52, red) and costained with LAMP1 (MAb ID4B, green). (D) Percentage of positively labeled parasites in naive BMDMs at 2 h postinfection. Values represent means ± standard errors of the means from three independent experiments. Representative images to the right showing parasites stained with SAG1 (MAb DG52, red in top panels) or GRA7 (rabbit Pc, green in bottom panels) and costained with host cell markers LAMP1 (MAb ID4B, green), LAMP2 (rat MAb GL2A7, green), autophagy adapter p62 (guinea pig Pc, red), or ubiquitin FK2 (MAb FK2, red). Bars, 5 µm.

Macrophages pretreated with IFN-γ/lipopolysaccharide (LPS) are well known to clear intracellular type III parasites efficiently (26). In order to test their innate ability to control intracellular T. gondii infection in the absence of IFN-γ, unactivated macrophages were infected with type I (GT1 strain) or type III (CTG strain) parasites at a multiplicity of infection (MOI) of 0.5. The cells were fixed at 30 min and 20 h postinfection, and the percentage of infected cells was calculated by automated plate-based imaging to acquire data from many independent microscopic fields (see Fig. S1 in the supplemental material). The percentage of CTG-infected RAW 264.7 macrophages was significantly reduced by >50% at 20 h compared to 30 min postinfection, whereas the percentage of GT1-infected macrophages was unaffected (Fig. 1A). We observed a similar enhanced clearance of the CTG strain in naive bone marrow-derived macrophages (BMDMs), while GT1 survived significantly better (Fig. 1B). This finding is somewhat surprising, as T. gondii is generally regarded as a pathogen that survives in naive macrophages, owing to its ability to actively invade the host cell and avoid phagocytic responses (4).

10.1128/mBio.01393-18.1 FIG S1  Pipeline used for automated plate-based image acquisition in Cytation3 imager. Raw TIFF files in different emission channels were acquired and imported into CellProfiler 2.1.1 (76) (downloaded from http://cellprofiler.org/releases/). Images were analyzed using custom-designed CellProfiler project template (the file for this custom script [Percentinfection.cpproj] is available on request). For measuring CellROX intensities, the Cy5 channel images were first illumination corrected before analysis. Download FIG S1, TIF file, 0.8 MB. Copyright © 2018 Matta et al. 2018 Matta et al. This content is distributed under the terms of the Creative Commons Attribution 4.0 International license.

Toxoplasma gondii is known to actively invade host cells, including macrophages, and to avoid lysosomal fusion (7). Hence, we considered that this difference in survival might reflect altered uptake and delivery of parasites to lysosomes. LAMP1 recruitment to parasites was measured to compare relative extents of active invasion and phagocytosis of CTG parasites in macrophages, as described previously (45). LAMP1 recruitment to vacuoles containing live parasites, representing active invasion, was much less than recruitment to those containing heat-killed, phagocytosed parasites for both GT1 and CTG strains, suggesting that the clearance of CTG was not due to phagocytosis in naive BMDMs (Fig. 1C).

Autophagy has also been implicated in growth restriction of susceptible T. gondii strains, albeit in IFN-γ-activated HeLa cells (23). Parasites targeted for destruction by this pathway become ubiquitinated, recruit autophagy adapters, and are engulfed by LC3 (23). Hence, we considered that a similar pathway might be triggered in naive macrophages infected with CTG. However, ubiquitination of the vacuole membrane as detected by the antibody FK2, which recognizes mono- and polyubiquitinated proteins, and recruitment of the LC3 adapter p62 were very low on both parasite strains in naive BMDMs (Fig. 1D). Additionally, the autophagy-associated marker LAMP2 was not elevated on CTG-containing vacuoles in naive BMDMs (Fig. 1D). Taken together, these findings demonstrate that the susceptibility of type III parasites in naive macrophages is not due to enhanced autophagy or lysosomal clearance.

**FIG 2:**
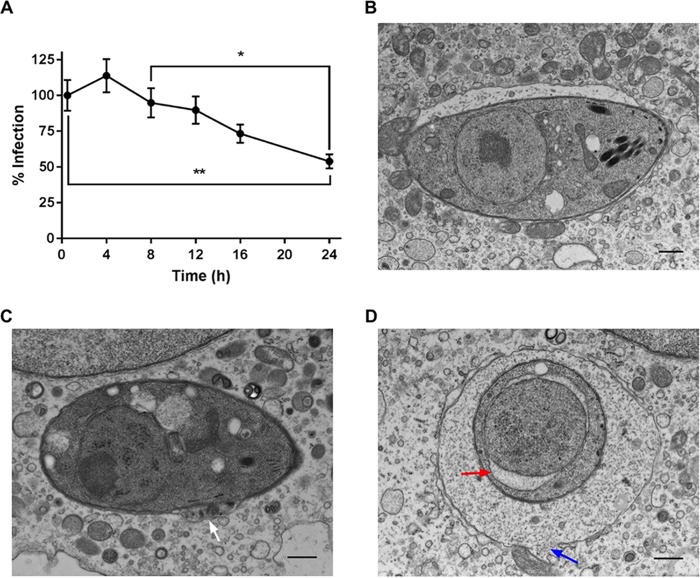
Kinetics of CTG strain parasite clearance. (A) Percentage of naive BMDMs that remained infected at 4, 8, 12, and 24 h compared to 0.5 h. Values represent means ± standard errors of the means for percentage of infected cells compared to 0.5 h with at least 100 fields per time point. One-way ANOVA with Tukey’s multiple comparison between the time points was used to test differences in infection. * and ** indicate significant differences at *P* < 0.05 and *P* < 0.01, respectively. (B to D) Transmission electron microscopy of CTG parasites in naive BMDMs sampled at intervals from 2 to 12 h postinfection. (B) Normal parasite with the parasitophorous vacuole. (C) Damaged parasite showing loss of the parasitophorous vacuole membrane and blebbing of the parasite membranes (white arrow). (D) Disrupted parasitophorous vacuole (blue arrow) and parasite with swollen nuclear envelope (red arrow). Bars, 500 nm.

Previous studies on the clearance of T. gondii in IFN-γ-activated macrophages have demonstrated that the clearance mediated by IRGs and GBPs occurs in the first few hours after infection (26, 31). As such, we examined the kinetics of clearance of CTG parasites in naive BMDMs. We observed that the percentage of infected macrophages started to decrease from 6 to 8 h postinfection and decreased linearly up to 24 h (Fig. 2A). As such, the kinetics of clearance differs substantially from previously characterized pathways. We also examined the morphology of the parasitophorous vacuole membrane, as previous studies in IFN-γ-activated cells have described a prominent scalloped appearance of the membrane, prior to rupture, in a process that is medicated by IRGs and GBPs (24, 26, 31, 46). A majority of parasitophorous vacuoles at early time points revealed normal cellular architecture of both the vacuole membrane and the parasite residing within (Fig. 2B). Over time, there was an accumulation of parasites that showed damage, including loss of the parasitophorous vacuole envelope (Fig. 2C), blebbing of parasite surface membranes (Fig. 2C), rupture of the parasitophorous vacuolar membrane with the host cytosol filling the space (Fig. 2D), and swelling of internal parasite membranes such as the nuclear envelope (Fig. 2D). However, at no stage did we observe the scalloped appearance of the parasitophorous vacuole membrane that accompanies Irg-mediated clearance.

**FIG 3:**
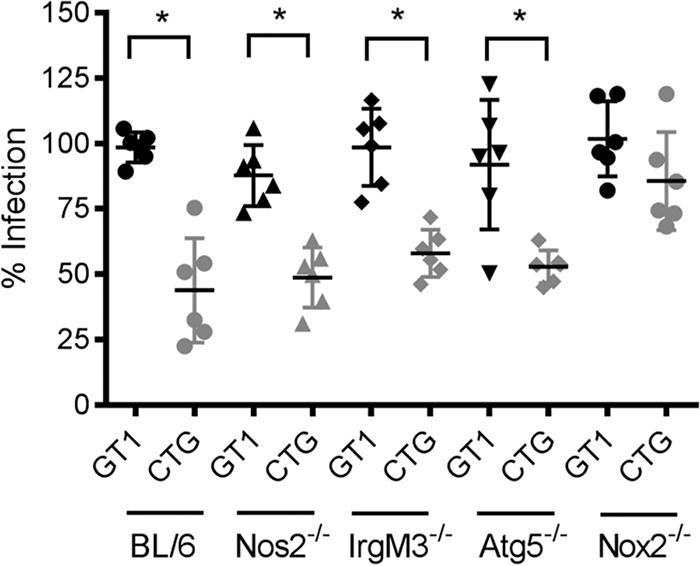
Survival of CTG parasites in bone marrow-derived macrophages. (A) Percentage of BMDMs from wild-type or mutant mice that remained infected at 20 h compared to 0.5 h postinfection. BL/6 represents wild-type C57BL/6 mice. All other mutants are on a similar background. Atg5^−/−^ represents Atg5^f/f^ crossed with LysMCre mice. Each symbol represents an independent biological experiment. Median values are marked with horizontal bars. Statistical analysis performed using Kruskal-Wallis test with Dunn’s correction for multiple tests. *, *P* ≤ 0.05.

In order to elucidate the mechanism for innate clearance of CTG in naive macrophages, BMDMs from different mutant mice lacking factors involved in intracellular pathogen clearance were examined. Naive BMDMs from mutant mice were infected with CTG strain parasites *in vitro*, and the survival at 20 h was assessed relative to infection at 0.5 h, as described above. Naive BMDMs lacking nitric oxide production (Nos2^−/−^), Irg-mediated defense (IrgM3^−/−^), or autophagy protein Atg5 (Atg5^f/f^ LysMCre) failed to reverse the clearance of CTG strain parasites (Fig. 3). These findings indicate that nitric oxide production, Irg-mediated defenses, and Atg-related pathways are unlikely to explain the increased susceptibility of CTG in naive mouse macrophages. However, BMDMs lacking gp91 (Nox2^−/−^; NADPH oxidase) showed significant loss of CTG clearance compared to wild-type cells (Fig. 3). The rescue of CTG parasites in Nox2^−/−^ macrophages suggested that their clearance in naive macrophages is dependent on reactive oxygen species (ROS) induction.

### NADPH oxidase mediated ROS production in T. gondii-infected macrophages.

**FIG 4:**
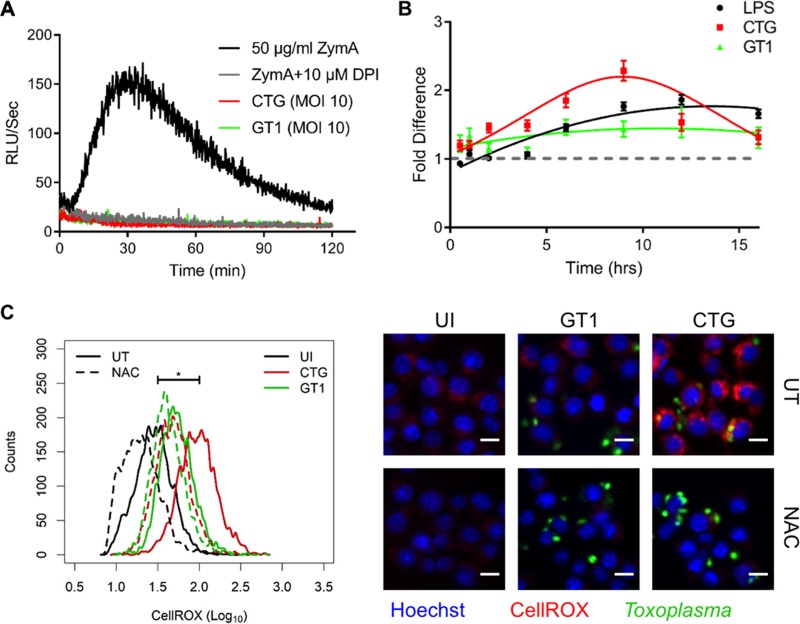
Induction of reactive oxygen species (ROS) during T. gondii *i*nfection. (A) Luminol detection of ROS activity from RAW 264.7 macrophages. Samples were incubated with zymosan A (50 µg/ml, black) or zymosan A with diphenyleneiodonium (DPI, 10 µM) (gray) or infected with CTG parasites (red) or GT1 parasites (green) at an MOI of 10. (B) CellROX-based intracellular detection of ROS following treatment of RAW 264.7 macrophages with LPS (100 µg/ml, black) or infection with CFSE-labeled CTG (red) or GT1 (green) parasites (MOI of 3). Cells were stained with CellROX (5 µM) for 30 min before fixation in 4% formaldehyde and imaging on Cytation3. Values represent mean intracellular CellROX intensity (Cy5 channel) ± standard errors of the means from at least 1,500 cells per sample normalized to uninfected/untreated cells (gray line) at each respective time point. (C) Histogram analysis of CellROX intensity (log_10_) in RAW 264.7 macrophages that were uninfected (UI, black), or infected with CFSE-labeled CTG (red, MOI of 3) or GT1 (green, MOI of 3) parasites and sampled at 9 h postinfection. For comparison, untreated (UT, solid) and NAC-pretreated (2 mM NAC, dashed) RAW 264.7 macrophages are shown. Representative images are shown to the right depicting CellROX (Cy5, red), parasites (CFSE, green), and nuclei (Hoechst stain, blue). Unpaired Student’s *t* test was used to compare ROS levels in uninfected and CTG-infected RAW 264.7 macrophages. *, significant difference between samples at *P* < 0.05. Bars, 10 µm.

NADPH oxidases in phagocytes are well known to produce ROS as a defense mechanism against invading pathogens (47). Although invasion of human macrophages by T. gondii proceeds without a ROS burst (5), infection of mouse macrophages with T. gondii has been shown to produce early ROS (48). Therefore, we tested whether CTG versus GT1 infection resulted in elevated ROS production in naive macrophages using luminol-based chemiluminescence. As expected, neither CTG nor GT1 produced ROS as defined by classical respiratory burst in naive RAW 264.7 macrophages (Fig. 4A) or BMDMs (Fig. S2). In contrast, incubation of macrophages with zymosan A (ZymA; 50 µg/ml), which acts as a Toll-like receptor 2 (TLR2) agonist, produced the expected ROS burst within a few minutes of incubation (Fig. 4A and S2). Treatment of macrophages with diphenyleneiodonium (DPI; 10 µM), which acts as an inhibitor of Nox assembly, abrogated the increase observed with ZymA treatment (Fig. 4A and S2), indicating that the observed ROS burst was Nox dependent. These results demonstrate that T. gondii does not trigger activation of the Nox complex in naive mouse macrophages during the initial stages of invasion.

10.1128/mBio.01393-18.2 FIG S2  Luminol-based ROS measurement from BMDMs incubated with zymosan A (50 µg/ml, black) or zymosan A with DPI (10 µM, gray) or infected with CTG (red) or GT1 (green) strain parasites at an MOI of 10. The example shown is a single representative experiment of more than 4 replicates with similar outcomes. Download FIG S2, TIF file, 0.2 MB. Copyright © 2018 Matta et al. 2018 Matta et al. This content is distributed under the terms of the Creative Commons Attribution 4.0 International license.

To test whether infection induced delayed production of intracellular ROS, we infected RAW 264.7 macrophages with carboxyfluorescein succinimidyl ester (CFSE)-labeled parasites and monitored intracellular ROS levels by staining with CellROX deep red. CTG-infected macrophages showed a time-dependent increase in cellular ROS levels that were elevated compared to GT1 (Fig. 4B). Treatment of RAW 264.7 macrophages with LPS (100 µg/ml, as a positive control) showed similar increases in ROS levels with increasing time (Fig. 4B). To confirm that the increase in CellROX staining was due to ROS, RAW 264.7 macrophages were pretreated with 2 mM *N*-acetylcysteine (NAC) and then infected with T. gondii. Pretreatment of macrophages with NAC, which acts as a cellular antioxidant, led to significant decrease of ROS levels in CTG-infected cells at 9 h postinfection, confirming that the increase in CellROX staining was due to ROS (Fig. 4C).

### Nox1^−/−^ and Nox2^−/−^ mice are susceptible to CTG infection.

**FIG 5:**
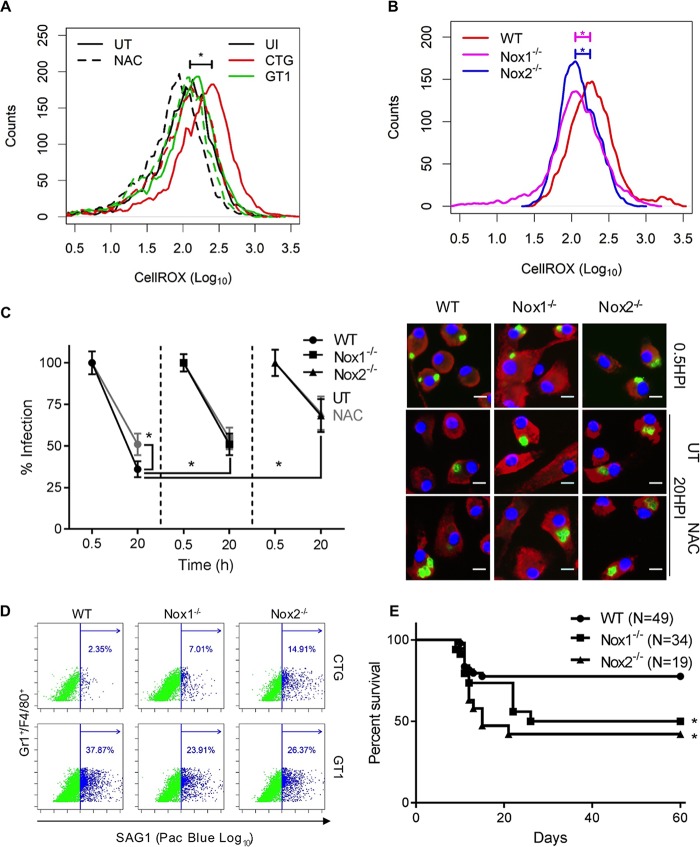
ROS-mediated clearance of T. gondii. (A) Histogram of CellROX intensity (log_10_) in BMDMs that were uninfected (UI, black) or infected with CTG (red, MOI of 3) or GT1 (green, MOI of 3) parasites and analyzed at 9 h postinfection. For comparison, untreated (UT, solid) and NAC-pretreated (2 mM NAC, dashed) BMDMs are shown. (B) ROS levels detected by CellROX staining of CTG-infected wild-type (WT, red), Nox1^−/−^ (magenta), or Nox2^−/−^ (blue) BMDMs. Unpaired Student’s *t* test was used to compare ROS levels in CTG-infected wild-type and Nox1^−/−^ or Nox2^−/−^ BMDMs. *, significant difference between samples at *P* < 0.05. (C) Percentage of cells remaining infected at 20 h compared to 0.5 h postinfection. BMDMs from wild-type (WT, ●), Nox1^−/−^ (■), and Nox2^−/−^ (▲) mice were infected with CTG parasites and left untreated (UT, black) or treated with NAC (2 mM *N*-acetylcysteine, gray). Values represent means ± standard errors of the means from three independent replicates with at least 50 fields per replicate. Representative images to the right are showing nucleus stained with Hoechst stain (100 ng/ml), parasites stained with SAG1 (MAb DG52, green), and host cells stained with LAMP1 (MAb 1D4B, red). Bar, 10 µm. *, significant difference between compared groups at *P* < 0.05 using unpaired Student’s *t* test. (D) Survival of CTG or GT1 parasites (detected with SAG1 labeled with Pac Blue) in inflammatory macrophages detected with Gr1^+^ and F4/80^+^. Wild-type (WT), Nox1^−/−^, and Nox2^−/−^ mice were infected with 10^6^ parasites by i.p. injection, and cells were harvested 2 days later for staining. (E) Kaplan-Meier survival curve of wild-type (WT) (*n =* 49), Nox1^−/−^ (*n* = 34), and Nox2^−/−^ (*n =* 19) mice. Mice were infected with 10^4^ CTG tachyzoites i.p. Log rank and Gehan-Breslow-Wilcoxon tests were used to compare survival of wild type (WT) with that of Nox1^−/−^ or Nox2^−/−^ mice. *, significant difference between compared groups at *P* < 0.05.

Our findings thus far suggest that increased clearance of CTG is due to elevated ROS production. To confirm this model, we were interested in testing whether the reversal of CTG clearance in Nox2^−/−^ macrophages as shown in Fig. 3 was due to a decrease in ROS production. First, we established that ROS production upon CTG infection was also evident in wild-type BMDMs at 9 h postinfection and that it was reversed in NAC-pretreated macrophages (Fig. 5A). Consistent with the above-described model, ROS levels produced in CTG-infected Nox1^−/−^ and Nox2^−/−^ macrophages were significantly lower than what was observed in wild-type cells (Fig. 5B). This finding suggested that both Nox isoforms are involved in ROS production in BMDMs infected with CTG. Consistent with this finding, CTG infection was significantly rescued in NAC-pretreated wild-type macrophages (Fig. 5C). Moreover, clearance of CTG was also significantly decreased in both Nox1^−/−^ and Nox2^−/−^ macrophages compared to wild-type cells (Fig. 5C). Nonetheless, these findings demonstrate that CTG infection of naive macrophages leads to increased intracellular ROS that depends on both Nox1 and Nox2 isoforms that contribute to parasite clearance.

The loss of CTG clearance in Nox-deficient macrophages *in vitro* suggested that this response might also be responsible for control of parasites *in vivo*. To examine this possibility, wild-type C57BL/6, Nox1^−/−^, and Nox2^−/−^ mice were challenged by intraperitoneal (i.p.) infection and monitored over time. Infection in Gr1^+^-F4/80^+^ cells, which represent inflammatory macrophages recruited during the early phase of infection (49), was monitored by flow cytometry. There was a significant increase of 3- to 7-fold in the percentage of CTG-infected Gr1^+^-F4/80^+^ cells in Nox1^−/−^ and Nox2^−/−^ mice compared to wild type (Fig. 5D), consistent with these mutants being less able to control early infection. In contrast, there was a higher percentage of GT1-infected Gr1^+^-F4/80^+^ cells, consistent with the intrinsic virulence of this strain (26), and this decreased only slightly in mutant mice compared to wild type, likely due to a higher influx of macrophages. The increased expansion of CTG in inflammatory macrophages from Nox-deficient mice at early time points also led to different outcomes of infection. Notably, Nox1^−/−^ and Nox2^−/−^ mice were significantly more susceptible than the wild-type mice to i.p. infection with CTG as shown by decreased survival (Fig. 5E). Brains were harvested from surviving mice after 60 days of infection for evaluation of chronic infection. However, the cyst burden was below the detection threshold limit of ~25 cysts/mouse brain in wild-type, Nox1^−/−^, and Nox2^−/−^ mice.

### Gbp5 contributes to CTG clearance in naive macrophages.

**FIG 6:**
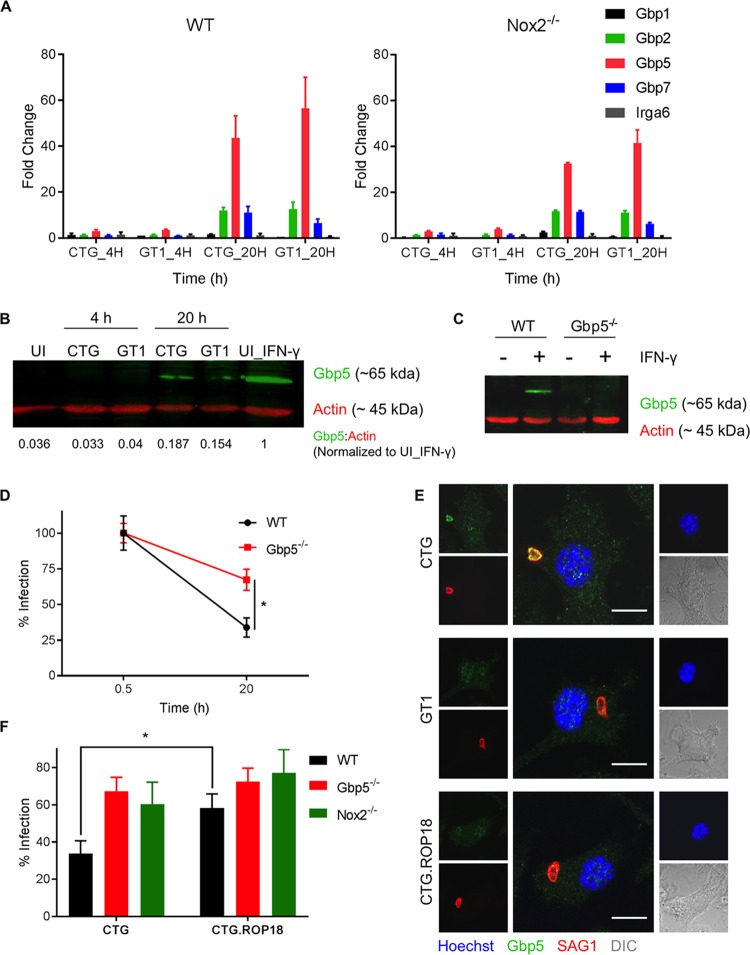
Gbp5 contributes to CTG clearance in naive macrophages. (A) Real-time PCR showing fold induction of mRNA transcripts in wild-type (WT) and Nox2^−/−^ BMDMs infected with CTG or GT1 at 20 h compared to uninfected cells at 4 h. Comparative *C*_*T*_ values were used to evaluate fold change in transcripts using actin as an internal transcript control. (B) Western blot of cell lysates from uninfected (UI) or T. gondii (CTG or GT1)-infected naive BMDMs (4 and 20 h) probed for Gbp5 (green) and actin (red). Normalized band intensities (numbers below the gel) are shown compared to uninfected IFN-γ (100 U/ml, 16 h)-stimulated BMDMs (lane 6). (C) Western blot of cell lysates of untreated or IFN-γ (100 U/ml, 16 h)-treated wild-type (WT) or Gbp5^−/−^ BMDMs probed for Gbp5 (green) and actin (red). (D) Percentage of wild-type (WT) and Gbp5^−/−^ BMDMs infected at 20 h compared to 0.5 h. *, significant difference at *P* < 0.05 between CTG-infected wild-type and Gbp5^−/−^ BMDMs at 20 h using Student’s *t* test. (E) Recruitment of Gbp5 in wild-type BMDMs infected with CTG, GT1, or CTG expressing ROP18 (CTG.ROP18) at 9 h postinfection. Cells were stained for Gbp5 (rabbit Pc, Gbp5, green), T. gondii (MAb DG52, SAG1, red), and nuclei (Hoechst stain, 100 ng/ml, blue). Bar, 10 µm. DIC, differential inference contrast. (F) Infection of wild-type (WT), Nox2^−/−^, and Gbp5^−/−^ BMDMs infected with CTG or ROP18-expressing CTG (CTG.ROP18). Percentage of wild-type (WT, black bars), Gbp5^−/−^ (red bars), and Nox2^−/−^ (green bars) macrophages infected at 20 h compared to 0.5 h. Values represent means ± standard errors of the means from at least three independent biological replicates. *, significant difference at *P* < 0.05 between compared groups using Student’s *t* test.

Although the findings above point to a role for ROS, this pathway only partially explains the defect in CTG as NAC treatment of either Nox1^−/−^ or Nox2^−/−^ mutants did not lead to additive effects (Fig. 5C). As such, we sought other explanations for the enhanced susceptibility of CTG in naive macrophages. Induction of Gbp1 (31) and Gbp2 (32) during T. gondii infection is associated with their recruitment onto the PVM and parasite damage, albeit best characterized in IFN-γ-activated macrophages. However, some Gbps, including Gbp3 and Gbp5, can be expressed in macrophages to a significant level independent of IFN-γ (32). Therefore, we tested whether any of these factors are induced upon T. gondii infection of macrophages independent of IFN-γ. To our surprise, BMDMs infected with either CTG or GT1 showed significant increase in expression of Gbp2, Gbp5, and Gbp7 mRNA at 20 h postinfection compared to uninfected cells (Fig. 6A). In particular, Gbp5 mRNA increased up to ~40-fold upon T. gondii infection, whereas Gbp2 and Gbp7 mRNA showed an ~10-fold increase (Fig. 6A). Gbp5 protein levels were also increased upon CTG or GT1 infection of naive macrophages, although to a lesser extent than IFN-γ treatment in wild-type BMDMs (Fig. 6B). The specificity of the ~65-kDa band of Gbp5 was confirmed by its absence in cell lysates of Gbp5^−/−^ BMDMs (Fig. 6C).

Previous studies have implicated Gbp5 in promoting NLRP3 inflammasome assembly and control of intracellular bacteria (33). ROS production has also been implicated in the NLRP3 inflammasome activation (50), although the NADPH oxidase complex is not required for inflammasome activation in response to strong agonists like nigericin or ATP (51). Therefore, we tested whether inflammasome activation occurs upon T. gondii infection of naive macrophages. Initially, we monitored IL-1β transcripts and found that infection with T. gondii only slightly altered their levels, while treatment with LPS greatly increased mRNA levels (Fig. S3A). We then measured IL-1β release to the supernatant as a marker of NLRP3 inflammasome activation. There was no significant increase in IL-1β secretion of either CTG- or GT1-infected naive macrophages (Fig. S3B). In contrast, LPS-primed macrophages did show significant secretion of IL-1β upon infection with either CTG or GT1 parasites by 20 h postinfection (Fig. S3B). Release of IL-1β was dependent on both LPS pretreatment and infection, consistent with the two-step hypothesis for NLRP3 inflammasome activation (52). The increase was significantly reduced upon NAC pretreatment of wild-type macrophages or in Gbp5^−/−^ and Nox2^−/−^ macrophages (Fig. S3B). Caspase-1 activation and its release into the supernatant are another marker for inflammasome activation. However, we also did not observe activated caspase-1 (cleaved 20-kDa caspase-1 fragment) in the supernatant of CTG- or GT1-infected naive or LPS-primed macrophages (Fig. S4). As expected, treatment with nigericin was able to elicit caspase-1 release in LPS-activated macrophages, and this effect was partially abrogated in Gbp5^−/−^ macrophages (Fig. S4). Collectively, these findings indicate that infection with T. gondii alone is not sufficient to induce inflammasome activation because priming signals are needed for IL-1β mRNA expression, but when combined with LPS, it provides a signal that drives IL-1β release. Although this pathway is partially dependent on Gbp5 and ROS, it did not lead to cell death (Fig. S5).

10.1128/mBio.01393-18.3 FIG S3  Induction and release of IL-1β. (A) Real time-quantitative PCR analysis of mRNA levels of IL-1β transcript in naive (UT; untreated) or LPS-preactivated (LPS) BMDMs. Cells were infected with CTG or GT1 strain parasites, and transcript levels were measured at 20 h postinfection. The mRNA levels were quantified with respect to actin as an internal control. The transcript levels are shown as fold difference compared to uninfected (UI) and untreated (UT) BMDMs. Values represent mean ± SD for an experiment done in triplicate. (B) Detection of IL-1β levels in supernatants by ELISA. Wild-type control (WT) or *N*-acetylcysteine-pretreated (NAC, 2 mM), Nox2^−/−^, or Gbp5^−/−^ BMDMs were cultured untreated (UT) or induced by LPS (10 ng/ml) for 24 h. Cells were either uninfected (UI) or infected with CTG or GT1 at an MOI of 3, and supernatants were collected at 20 h postinfection for ELISA. Values represent mean ± SD for an experiment done in triplicate. *, significant difference at *P* < 0.05 between compared groups using Student’s *t* test. Download FIG S3, TIF file, 0.2 MB. Copyright © 2018 Matta et al. 2018 Matta et al. This content is distributed under the terms of the Creative Commons Attribution 4.0 International license.

10.1128/mBio.01393-18.4 FIG S4  Release of cleaved caspase detected by Western blotting. Immunoblot for caspase-1 in supernatants of LPS (10 ng/ml)-preactivated wild-type and Gbp5^−/−^ BMDMs infected with CTG or GT1 for 20 h. Nigericin treatment (10 µM for 3 h) (NIG) was used as a positive control. The levels of cleaved caspase-1 were normalized to level of total protein loaded per well. A duplicate gel was stained with Coomassie blue for reference (lower panel). Values below the immunoblot correspond to the levels of caspase-1 in the supernatant normalized to amount loaded and compared to uninfected (UI) wild-type BMDMs. Download FIG S4, TIF file, 0.3 MB. Copyright © 2018 Matta et al. 2018 Matta et al. This content is distributed under the terms of the Creative Commons Attribution 4.0 International license.

10.1128/mBio.01393-18.5 FIG S5  Cell survival following infection. (A) Quantification of the percentage of dead cells (ethidium homodimer-1 positive) in untreated (UT) or LPS-preactivated (LPS, 10 ng/ml) wild-type (WT) and Gbp5^−/−^ BMDMs infected with CTG or GT1 for 20 h. Treatment with 0.1% saponin for 10 min was used as a positive control. (B) Representative images for LPS-activated wild-type (WT) and Gbp5^−/−^ BMDMs either untreated (UT) or nigericin treated (10 µM for 3 h). Calcein stains live cells (green), while ethidium homodimer stains nuclei of dead cells (red). Bar, 10 µm. Values represent mean ± SD of percentage of dead cells measured in triplicate. *, significant difference at *P* < 0.05 between compared groups using Student’s *t* test. Download FIG S5, TIF file, 0.6 MB. Copyright © 2018 Matta et al. 2018 Matta et al. This content is distributed under the terms of the Creative Commons Attribution 4.0 International license.

Given the absence of evidence for inflammasome-mediated clearance, we sought evidence that Gbp5 might be directly involved in mediating clearance of CTG in naive cells. Interestingly, CTG clearance in Gbp5^−/−^ macrophages was significantly reversed (Fig. 6D). Despite evidence that Gbp5 contributes to clearance, we observed only modest evidence for its recruitment to parasitophorous vacuoles containing CTG (3.1% ± 2.4% [mean ± standard deviation {SD}, *n* = 4] at 9 h postinfection) in naive macrophages (Fig. 6E). However, Gbp5 recruitment was absent (0%) in either GT1 or CTG parasites expressing the type I ROP18 allele (CTG.ROP18) (Fig. 6E). Moreover, the clearance of the CTG.ROP18 strain was also significantly less than that of CTG in wild-type BMDMs and was similar to clearance of CTG in Gbp5^−/−^ BMDMs (Fig. 6F). These findings suggested that ROP18 expression in type I parasites is involved in resisting Gbp5-mediated clearance in naive macrophages to *Toxoplasma* infection and that Gbp5 can exert its effects in part via mechanisms other than targeting the PV.

## DISCUSSION

Toxoplasma gondii is known for its ability to enter and survive in phagocytic cells where it avoids lysosome fusion and replicates within a sequestered vacuole. Surprisingly, we found that highly avirulent type III strains are rapidly cleared in naive macrophages, even in the absence of interferon activation. Clearance was associated with gradual disruption of the PV and damage to the parasites in a process that did not involve lysosome fusion, recruitment of autophagy adapters, or vesiculation mechanisms that have been described previously. Additionally, there was no effect on parasite clearance in the absence of different host factors like Atg5, Nos2, or Irgs that are involved in IFN-γ-mediated growth restriction and killing of T. gondii in murine macrophages. Instead, naive macrophages cleared avirulent type III T. gondii parasites via induction of cellular ROS. Disruption of the NADPH oxidase complex in Nox1- or Nox2-deficient cells partially reversed the clearance defect. Nox1- and Nox2-deficient mice were also more susceptible to infection, indicating that this mechanism partially accounts for avirulence of type III strains *in vivo*. In addition, infection of macrophages leads to upregulation of GBPs, and the clearance of type III parasites was partially reversed in Gbp5^−/−^ macrophages. Although both NADPH oxidase and Gbp5 have previously been implicated in activation of the NLRP3 inflammasome, infected cells did not undergo caspase-1 cleavage or pyroptosis. These findings highlight new roles for the NAPH oxidase and GBPs in control of intracellular pathogens via pathways intrinsic to naive cells that do not require prior activation by interferons.

Invasion of T. gondii into macrophages results in formation of a parasitophorous vacuole that fails to acquire markers of endosomes and also does not fuse with lysosomes (45). This outcome can be modulated by adding antibody to opsonize the parasite for internalization via Fc receptors (3). Interestingly, the machinery to drive lysosome fusion is also found in nonphagocytic cells as shown by expression of Fc receptors, which is sufficient to drive opsonized T. gondii to lysosomes (53). The ability of T. gondii to avoid lysosome fusion was not responsible for the defect in type III strain survival and is likely universal to all strains. Additionally, we did not observe an increase in ubiquitination or recruitment of autophagy adapters that have been linked to control of intracellular bacterial by a process termed xenophagy (54–56). Instead, the PV formed normally but then underwent gradual disruption characterized by loss of integrity of the vacuolar membrane, damage to parasite membrane, and degradation of internal membrane structures. These features are very different from the scalloped appearance of the parasitophorous vacuole membrane that accompanies clearance in IFN-γ-activated cells, a process linked to recruitment of Irgs (26). The recruitment of Irgs to the PVM is dependent on a noncanonical autophagy pathway and is disrupted in Atg5^−/−^ cells and other components of the core pathway (46, 57). Consistent with a lack of evidence for PVM vesiculation, the enhanced clearance of type III parasites was unaltered in Atg5^−/−^, IrgM3^−/−^, or Nos2^−/−^ cells. Although the precise reason for loss of type III parasites is uncertain, it may involve damage from ROS and/or transient association with Gbp5, as discussed below. Regardless of the precise mechanism of membrane damage, this outcome differs morphologically and kinetically from previously described pathways for clearance of T. gondii in macrophages.

ROS induction in naive macrophages is associated with production of a respiratory burst, which is a rapid and elevated increase in production of superoxide free radicals and H_2_O_2_ upon activation of NADPH oxidases in response to early recognition of pathogens (58). Reversal of CTG clearance in Nox1^−/−^ or Nox2^−/−^ macrophages suggests that the parasites are cleared by induction of NADPH oxidase-mediated ROS in naive macrophages. However, we did not observe a classical respiratory burst, which typically is evoked very rapidly and reaches high levels, as seen with zymosan stimulation. Rather, ROS production following T. gondii infection was muted in its extent and delayed, with maximum levels detected at ~9 h postinfection. It remains possible that the role described here for NADPH oxidase could also be due to mitochondrial production of ROS, since previous studies have shown that these pathways work cooperatively in host defense against intracellular bacteria (59). Regardless of the exact mechanism of ROS production, the role of NADPH oxidase was also seen *in vivo* where both Nox1^−/−^ and Nox2^−/−^ mice showed increased infection of inflammatory macrophages during early infection with CTG, leading to increased susceptibility compared with wild-type mice. The reason for the enhanced induction of ROS by type III strains is uncertain, but numerous genetic differences occur between type I and III strains, including the polymorphic T. gondii protein GRA7 (17, 60), which has been shown to elicit Nox2-dependent ROS in BMDMs (61). Prior studies have also suggested a role for NADPH oxidase during acute infection of mice with type I parasites that elicited ROS production by infected myeloid inflammatory cells during 3 to 4 days postinfection (62). Additionally, preexposure to ATP can increase ROS upon type I T. gondii infection of macrophages (63). Collectively, these findings suggest that ROS production can lead to control of T. gondii under certain circumstances. Our findings indicate that type III strains induce significantly higher levels of ROS and that this contributes to their clearance in naive macrophages.

The observation that susceptible CTG parasites were only partially reversed in Nox1^−/−^ and Nox2^−/−^ macrophages suggested that some other factor also contributes to clearance of parasite in naive macrophages. We therefore examined the expression of other innate effectors that have been implicated in control of T. gondii, including IRGs and GBPs (64). Expression of Gbp5 was strongly induced upon infection with T. gondii, while Gbp2 and Gbp7 were induced at lower levels, and no substantial change was seen in Gbp1 or Irga6. Gbp5 is normally expressed in unstimulated mouse macrophages, and like other GBPs, it is also induced by interferon treatment (32). Induction of GBPs upon T. gondii infection occurred independently of addition of interferon, although the basis for this increased expression is uncertain. Unlike Gbp1 and Gbp2, Gbp5 has not previously been associated with increased recruitment to T. gondii-containing vacuoles in interferon-activated cells (28). Consistent with this observation, Gbp5 was detected on only a minority of CTG-containing vacuoles (i.e., ~3%) in naive macrophages, albeit at higher levels than seen in GT1-infected cells. Despite its not being strongly recruited to the vacuole, loss of Gbp5 in the knockout led to the increased survival of CTG parasites in naive macrophages. Hence, it is possible that Gbp5 plays a role in parasite restriction that is independent of stable association with the parasite vacuole. A similar finding has previously been reported for GBP1 in human cells, where parasite control occurs independently of recruitment to the vacuole in A549 cells (65). Alternatively, it is possible that recruitment of Gbp5 to T. gondii-containing vacuoles is transient or that the demise of the compartment is sufficiently rapid that it does not remain stably associated with the membrane. Consistent with the idea that Gbp5 acts proximal to the parasitophorous vacuole membrane, expression of ROP18 in the type III CTG strain reversed the enhanced clearance seen in naive macrophages. ROP18 has previously been shown to mediate resistance to Gbp1 in interferon-activated macrophages, and this protective effect is thought to occur at the parasitophorous vacuole membrane (31). Similarly, expression of ROP18 in CTG parasites may block the action of Gbp5, or an associated factor, on the parasitophorous vacuole membrane, thus protecting the parasite from damage. Distinguishing between these two mechanisms will require further study to define the molecular partners of Gbp5 and to ascertain whether ROP18 acts directly or indirectly to block this host effector.

Although both NADPH oxidase and Gbp5 were individually important in the control of CTG clearance in naive macrophages, our studies do not define whether these processes operate as separate parallel pathways or if they converge on a common mechanism. One common process that is influenced by both cellular ROS and Gbp5 is NLRP3 inflammasome activation (33, 50). Our findings are only partially consistent with activation of the inflammasome pathway, which normally requires separate priming and activation steps (52). Infection with type I or type III strain parasites did not drive IL-1β expression on its own, indicating that the parasites are not able to provide the normal priming step. However, infection with type I (GT1) or type III (CTG) strain parasites was able to induce IL-1β release from LPS-treated cells, indicating that infection can activate the NLRP3 inflammasome in cells that have previously been primed (52). However, we did not detect cleavage and release of caspase-1 or activation of pyroptosis in T. gondii-infected cells even following LPS priming, suggesting that infection with these strains does not result in classical inflammasome activation. Interestingly, in the present study both Nox2 and Gbp5 were required for optimal IL-1β release in LPS-primed and infected cells, consistent with previous findings that these processes regulate the NLRP3 inflammasome (33, 50). Thus, one model to explain the roles for NADPH oxidase and Gbp5 in restricting survival of type III strains of T. gondii is that they collectively activate an inflammasome-like process, albeit not one that leads to cell death. This observation agrees with several previous studies that used type II strain parasites to infect BMDMs *in vitro* and found that IL-1β gets secreted from primed cells without inducing pyroptosis (37, 38). Although engagement of the inflammasome complex does not appear to induce cell death in the murine system, it nonetheless has been shown to promote parasite control *in vivo*, an effect that may relate to processing of cytokines, including IL-1β (37) and IL-18 (38). Thus, the enhanced susceptibility of type III strains *in vivo* may stem from both the increased susceptibility to clearance in naive macrophage and downstream mediators that influence immunity.

Type III strains are highly susceptible to clearance by IRGs and GBPs in activated mouse macrophages, in part due to their lack of expression of the parasite effector ROP18 (26, 27). IRGs (66, 67) and GBPs (64) are strongly induced following exposure to IFN-γ, and they provide the major mechanism of resistance in the mouse following activation of the immune response. Collectively, the susceptibility of type III strains to clearance in naive cells, as reported here, and in IFN-γ-activated cells, as reported previously, contributes to their profound avirulence in the murine system. Type II strains, which have intermediate virulence in mice, were not susceptible to clearance in naive cells (S. K. Matta, unpublished data), although we have not examined the consequence of loss of ROP18 in this genetic background. Type III strains may also be susceptible to ROS in human cells, although infection of human monocyte-derived macrophages with type I strains of T. gondii has been reported to not trigger a strong respiratory burst (5). Moreover, chronic granulomatous disease (CGD) patients, who lack a functional PHOX oxidase complex, are not known to be more susceptible to toxoplasmosis (68), suggesting that there are other intrinsic mechanisms for control of this parasite in human cells. Defining the roles of various pathways in human cells, including the role of additional GBPs, in the control of T. gondii remains an important goal for future studies.

Although T. gondii is considered to be highly adapted to survive in phagocytic cells, including macrophages, our findings indicate that this is not a universal attribute. Rather, type III strains are readily cleared from naive macrophages, a property that may underlie their avirulence in mice, and possibly the rarity of infections caused in humans. We have identified Nox-mediated ROS generation and Gbp5 as novel factors involved in regulating intracellular survival of avirulent type III parasites within macrophages. These factors may be linked by a common involvement of the NLRP3 inflammasome, although restriction of parasite survival does not rely on activating pyroptosis. Together, these factors act independently of interferon activation, suggesting that they provide an autonomous system for cell-intrinsic control of intracellular infection.

## MATERIALS AND METHODS

### Reagents and antibodies.

Zymosan A (ZymA), DPI, luminol, horseradish peroxidase (HRP), lipopolysaccharide (LPS), and *N*-acetylcysteine (NAC) were obtained from Sigma (St. Louis, MO, USA). CellROX deep red, goat anti-mouse IgG, goat anti-rat IgG, goat anti-rabbit, or goat anti-guinea pig secondary antibodies conjugated to Alexa 594 or Alexa 488 were obtained from Life Technologies (Grand Island, NY, USA). Carboxyfluorescein succinimidyl ester (CFSE) was obtained from Thermo Fisher Scientific (Waltham, MA, USA). Rabbit polyclonal (Pc) anti-Gbp5 antibody was obtained from Proteintech Group (Rosemont, IL, USA). Cy5-labeled Gr1, fluorescein isothiocyanate (FITC)-labeled F4/80, and phycoerythrin (PE)-labeled B220 were obtained from BD Biosciences (San Jose, CA, USA). Mouse anti-actin (C4 clone) antibody was obtained from Millipore (MA, USA). Mouse monoclonal antibody (MAb) anti-caspase-1 (p20) was obtained from Adipogen Life Sciences (San Diego, CA). Goat anti-rabbit IgG IR800 and anti-mouse IgG IR700 were obtained from Li-Cor Biosciences (Lincoln, NE, USA). T. gondii parasites were stained with mouse MAb DG52 against the surface antigen SAG1 (69). MAb DG52 was labeled with Pacific Blue (Pac Blue) dye using a protein labeling kit (Invitrogen) to generate Pac Blue-labeled antibody for parasite detection by flow cytometry. GRA7 was detected using a rabbit Pc serum described previously (60). LAMP1 was localized with rat MAb ID4B, obtained from the Developmental Studies Hybridoma Bank (http://dshb.biology.uiowa.edu). Guinea pig polyclonal anti-p62 was obtained from Progen (Heidelberg, Germany). Mouse MAb FK2 against polyubiquitin and monoubiquitin was obtained from EMD Millipore Corporation (Billerica, MA). Anti-LAMP2 (rat MAb GL2A7) was obtained from Abcam. Fluorescein isothiocyanate (FITC)-conjugated Dolichos biflorus lectin (DBL) was obtained from Vector Laboratories (Burlingame, CA, USA).

### Parasite and macrophage culture.

Type I (GT1, ATCC 50853) and type III (CTG, ATCC 50842) strains of T. gondii were grown as tachyzoites in human foreskin fibroblasts (HFFs; obtained from the laboratory of John Boothroyd, Stanford University) as described previously (70). Parasites for all experiments were harvested shortly after natural egress, purified by passage through a 20-gauge needle, and separated from host cell debris using 3.0-µm polycarbonate Nuclepore filters (Whatman). RAW 264.7 macrophage cells (ATCC TIB-71) were maintained in Dulbecco’s modified Eagle’s medium (DMEM; Life Technologies) with 10% fetal bovine serum (FBS; Life Technologies) and cultured at 37°C and 5% CO_2_. Bone marrow-derived macrophages (BMDMs) were isolated from the femurs of adult mice, as described previously (71). BMDMs were harvested in DMEM containing 20% L929 conditioned medium, 10% FBS, and 5% horse serum in a 100-mm by 20-mm untreated polystyrene culture dish (Corning). After a week of culture, the cells were maintained in DMEM containing 10% L929 conditioned medium, 10% FBS, and 5% horse serum. For experiments, BMDMs were rinsed in calcium-magnesium-free phosphate-buffered saline (PBS), harvested by incubation with 1.25 mM trypsin for 20 min, and seeded in DMEM containing 10% FBS. All strains and host cell lines were determined to be mycoplasma negative using the e-Myco Plus kit (Intron Biotechnology).

### Animals.

Mice were housed and bred locally at Washington University in an Association for Assessment and Accreditation of Laboratory Animal Care-approved facility. Animal studies were conducted according to the U.S Public Health Service Policy on Humane Care and Use of Laboratory Animals.

All mice were on a C57BL/6 background. C57BL/6 wild-type, Nos2^−/−^, and Nox1^−/−^ mice were purchased from Jackson Laboratories. Nox2^−/−^ mice, also referred to as X-CGD or gp91^−/−^ mice (72), were provided by M. Dinauer, Washington University in St. Louis. Gbp5^−/−^ mice were provided by the laboratory of John D. MacMicking, Yale University School of Medicine. IrgM3^−/−^ mice (66) were obtained from Greg Taylor, Duke University. Atg5^f/f^ mice were crossed with LysMCre mice and genotyped as described previously (73, 74).

For survival assays, 8- to 12-week-old mice were injected with 10^4^ CTG tachyzoites i.p., and survival was monitored for 60 days. For *in vivo* parasite clearance, 10^6^ parasites were injected i.p. into 8- to 12-week-old mice. Mice were sacrificed 48 h postinfection, and peritoneal cells were isolated by lavage with ice-cold PBS. Cells were fixed with 4% formaldehyde and stained with FITC-F4/80 and Cy5-Gr1 to identify inflammatory macrophages, Pac Blue-DG52 to label parasites, and PE-B220 to gate out B cells. The fraction of parasite-positive Gr1^+^-F4/80^+^ cells was used to monitor the burden of parasite infection based on acquisition of 50,000 events per sample on a FACSCanto II flow cytometer at the Flow Cytometry research core, Department of Pathology and Immunology, Washington University in St. Louis. Data were analyzed using the flowCore R package (75).

### Chronic cyst burden estimation.

Surviving mice after 60 days of infection with the CTG strain were sacrificed, and their brains were harvested in 1 ml of 6% (vol/vol) formaldehyde and 0.25% (vol/vol) Triton X-100 in cold PBS. Brain homogenate was prepared by passing the tissue multiple times through a 16-gauge needle. The homogenates were then centrifuged at 400 × *g* for 10 min at 4°C. The pellet was then blocked with 10% (vol/vol) normal goat serum in PBS at 4°C for 1 h. The homogenate was then centrifuged at 400 × *g* for 10 min, and the pellet was resuspended with 20 µg/ml FITC-conjugated Dolichos biflorus lectin (DBL) in 10% goat serum for 2 h at room temperature. The homogenates were washed twice with 10% goat serum and resuspended in 1 ml PBS for microscopic examination. Each sample was examined using three aliquots of 12.5 µl each using an epifluorescence microscope equipped with an FITC emission channel.

### Immunofluorescence microscopy.

The cells were fixed in 4% formaldehyde for 20 min at room temperature, blocked using 5% FBS and 5% normal goat serum with 0.02% saponin in PBS for 30 min, and incubated with primary antibodies in 1% FBS with 0.02% saponin in PBS for 90 min. Cells were washed three times with PBS and incubated with Alexa-conjugated secondary antibodies and Hoechst stain (100 ng/ml) to stain nuclei (Life Technologies) for 30 min. Cells were then washed three times with PBS followed by image acquisition. Parasites and macrophages were labeled with 1:1,000 DG52 and rat 1:1,000 anti-LAMP1 as primary antibodies followed by 1:1,000 anti-mouse IgG-Alexa 488 and anti-rat IgG-Alexa 594 as secondary antibodies. Cells plated in 96-well µCLEAR black plates (Greiner Bio International) for *Toxoplasma* survival assay were imaged on a Cytation3 cell imaging multimode reader (BioTek) using a 20× objective (numerical aperture [NA], 0.45). Cells plated on coverslips for Gbp5 localization were imaged on an LSM880 confocal laser scanning microscope (Carl Zeiss) using a 63× objective (NA, 1.4) as part of the Microbiology Imaging Facility, Washington University in St. Louis.

### Transmission electron microscopy.

For ultrastructural analyses, samples were fixed in 2% paraformaldehyde-2.5% glutaraldehyde (Polysciences Inc., Warrington, PA) in 100 mM sodium cacodylate buffer, pH 7.2, for 2 h at room temperature and then overnight at 4°C. Samples were washed in sodium cacodylate buffer at room temperature and postinfection fixed in 1% osmium tetroxide (Polysciences Inc.) for 1 h. Samples were then rinsed extensively in distilled water (dH_2_O) prior to *en bloc* staining with 1% aqueous uranyl acetate (Ted Pella, Redding, CA) for 1 h. Following several rinses in dH_2_O, samples were dehydrated in a graded series of ethanol and embedded in Eponate 12 resin (Ted Pella Inc.). Sections of 95 nm were cut with a Leica Ultracut UCT ultramicrotome (Leica Microsystems Inc., Bannockburn, IL), stained with uranyl acetate and lead citrate, and viewed on a JEOL 1200 EX transmission electron microscope (JEOL USA Inc., Peabody, MA) equipped with an AMT 8-megapixel digital camera and AMT Image Capture Engine V602 software (Advanced Microscopy Techniques, Woburn, MA) as part of the Microbiology Imaging Facility, Washington University in St. Louis.

### *Toxoplasma* intracellular survival assay.

RAW 264.7 cells or BMDMs were seeded in 96-well µCLEAR black plates (Greiner Bio International) 24 h prior to infection. Cells were infected with CTG or GT1 at an MOI of 0.5 for 30 min followed by three PBS washes to remove extracellular parasites. Cells were fixed at 30 min and 20 h postinfection using 4% formaldehyde and stained to detect host cell (anti-LAMP1) and parasites (anti-SAG1) followed by Alexa-conjugated secondary antibodies. Images were acquired at 20× on the Cytation3 imager, and the percentage of infected cells per field was determined using CellProfiler 2.1.1 (see Fig. S1 in the supplemental material). For RAW 264.7 macrophages, the number of infected cells at each time point was used to calculate the percentage of infection. Data from at least 50 fields per experiment were used to calculate the percent decrease in infected cells at 20 h versus 30 min postinfection.

### Luminol-based respiratory burst assay.

One million RAW 264.7 cells or BMDMs per 100 µl of phenol-free DMEM were warmed to 37°C for 15 min. Luminol and horseradish peroxidase were added to cells at final concentrations of 200 µM and 20 U/ml, respectively, and incubated for 5 min at 37°C. The cells were then infected with parasites at an MOI of 10 or incubated with 50 µg/ml ZymA (with or without 10 µM DPI). Chemiluminescence was then recorded as relative light units (RLU) per second in real time for the next 2 h using the Cytation3 imager and analyzed using Gen5 software.

### Intracellular ROS measurement assay.

RAW 264.7 cells or BMDMs were seeded in 96-well µCLEAR black plates 24 h prior to infection. Parasites were labeled by incubation with 2.5 µM carboxyfluorescein succinimidyl ester (CFSE) for 5 min before infecting macrophages at an MOI of 2 for 30 min. Cells were then washed three times with PBS to remove extracellular parasites. At different time intervals postinfection, cells were stained with 5 µM CellROX deep red (Thermo Fisher Scientific) and Hoechst stain (100 ng/ml) for 30 min. After washing with PBS, images were acquired at 20× on the Cytation3 imager. After illumination correction of each image, integrated emission intensity per cell in the Cy5 channel was calculated for uninfected and infected cells across many fields. Histograms of the Cy5 intensity values were generated from at least 2,000 cells per sample.

### Western blotting.

Cell lysates of BMDMs were prepared using CellLytic M (Sigma) mixed with Complete Mini protease inhibitor cocktail (Roche). Cell supernatants were collected after centrifugation at 6,000 × *g* for 10 min at room temperature (RT) to avoid cell debris. Total protein was measured in each sample using the bicinchoninic acid (BCA) protein assay kit (Pierce, Thermo Fisher Scientific). Samples were boiled at 95°C for 15 min in Laemmli buffer containing 100 mM dithiothreitol (DTT). Samples were separated using SDS-PAGE and transferred onto a nitrocellulose membrane. The membrane was blocked in a 1:1 mixture of Odyssey blocking buffer (OBB; Li-Cor Biosciences) and PBS overnight at 4°C. The membrane was incubated with rabbit polyclonal anti-Gbp5 or anti-caspase-1 at 1:1,000 and mouse anti-actin (C4 clone; Millipore) at 1:4,000 for 2 h at RT in a 1:1 mixture of Odyssey blocking buffer and PBS with 0.1% Tween 20 (PBS-Tween). The blot was washed three times for 5 min each with PBS-Tween and incubated with anti-rabbit IgG IR800 and anti-mouse IgG IR700 at 1:15,000 for 2 h at RT in a 1:1 mixture of Odyssey blocking buffer and PBS-Tween. The blot was washed three times for 5 min each with PBS-Tween followed by infrared imaging on a Li-Cor Odyssey imaging system. Band intensities were calculated using the Odyssey software.

### Real-time PCR.

Samples were lysed, and RNA was extracted using the Qiagen RNeasy minikit per the manufacturer’s instructions. cDNA was prepared using the Bio-Rad iScript cDNA synthesis kit per the manufacturer’s instructions. Real-time PCR of all the genes was performed using Clontech SYBR Advantage qPCR premix per the manufacturer’s instructions. Data acquisition was done in QuantStudio3 (Applied Biosystems) and analyzed in QuantStudio design and analysis software (Applied Biosystems). Primers are listed in Table S1. Comparative threshold cycle (*C*_*T*_) values were used to evaluate fold change in transcripts using actin as an internal transcript control.

10.1128/mBio.01393-18.6 TABLE S1  Real-time PCR primers. Download TABLE S1, DOCX file, 0.02 MB. Copyright © 2018 Matta et al. 2018 Matta et al. This content is distributed under the terms of the Creative Commons Attribution 4.0 International license.

### ELISA for IL-1β.

Supernatants from samples were collected after centrifugation at 6,000 × *g* for 10 min at RT to avoid cell debris. The supernatants were stored at −80°C if not used immediately. IL-1β levels in the supernatant were measured using the mouse IL-1β/IL-1F2 DuoSet enzyme-linked immunosorbent assay (ELISA) kit (R&D Systems) per the manufacturer’s instructions.

### Cell viability staining.

BMDMs were seeded in 96-well µCLEAR black plates. Cells were either untreated or treated with 10 ng/ml of LPS for 24 h prior to infection or treatment. The cells were infected with CTG or GT1 parasites at an MOI of 3 for 20 h. Nigericin (10 µM) was used to treat cells for 3 h prior to fixation. As a positive control, 0.1% (wt/vol) saponin treatment for 10 min was used to lyse cells. The samples were washed once with PBS and stained with the Live/Dead staining kit (Thermo Fisher Scientific) containing a mixture of 2 µM calcein and 1 µM of ethidium homodimer-1 in PBS for 30 min. The cells were washed with PBS and fixed with 4% formaldehyde for 15 min. The cells were washed with PBS, imaged using a Cytation3 imager at 20×, and analyzed using CellProfiler 2.1.1.

### Statistical analyses.

Unpaired two-tailed Student’s *t* tests were used for comparison between experiments with normally distributed data using Prism (GraphPad). All experiments were performed at least three independent times, and statistical analyses were conducted on the composite data unless reported otherwise. One-way analysis of variance (ANOVA) with Tukey’s multiple-comparison test was used to compare CTG clearance kinetics in BMDMs. Non-normally distributed data were analyzed using the Kruskal-Wallis test with Dunn’s correction for multiple tests. Survival statistics were compared using log rank and Gehan-Breslow-Wilcoxon tests in Prism (GraphPad).
