# Supplementary figures and images for: NADPH Oxidase and Guanylate Binding Protein 5 Restrict Survival of Avirulent Type III Strains of Toxoplasma gondii in Naive Macrophages
Source: mBio. 2018 Aug 28;9(4):e01393-18. doi: 10.1128/mBio.01393-18 (PMC6113620; doi:10.1128/mBio.01393-18)

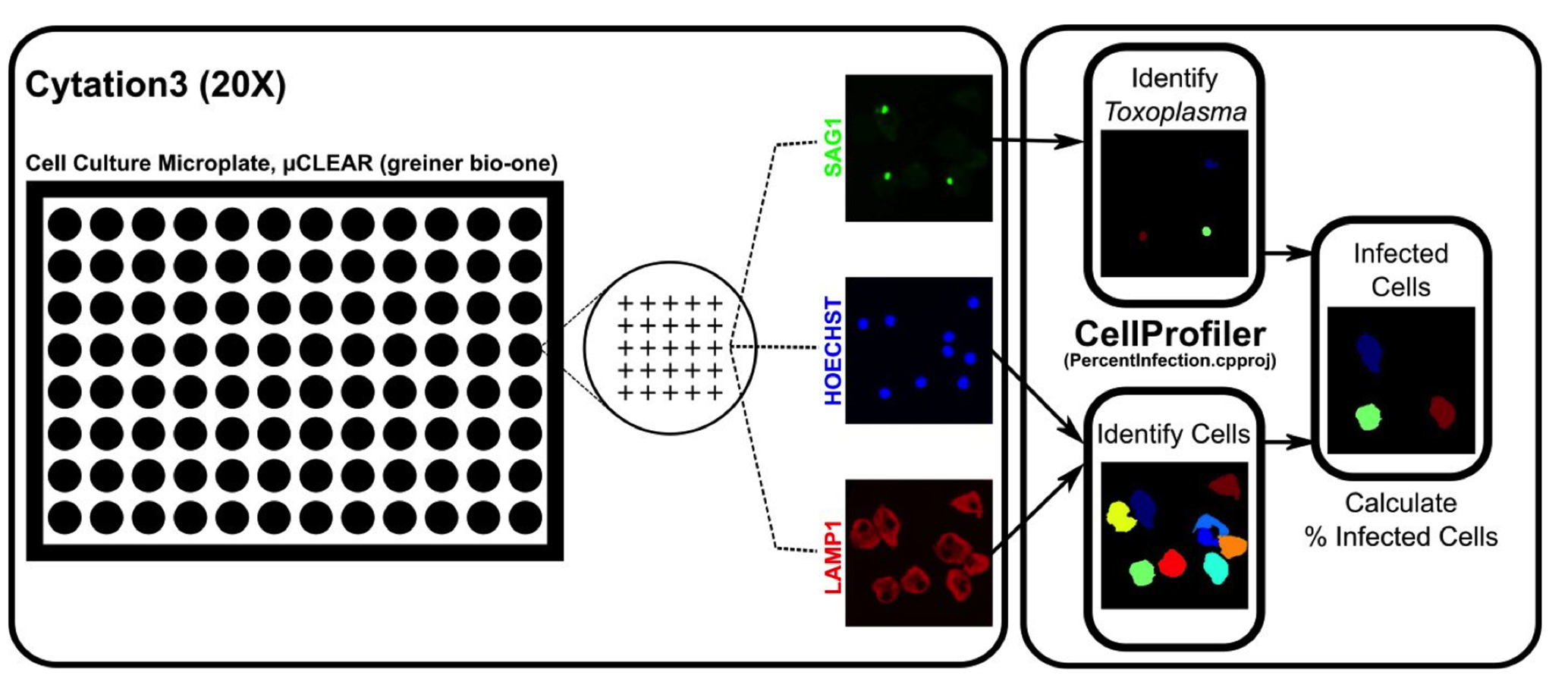

Supplement: FIG S1 [file mbo004184036sf1.tif]

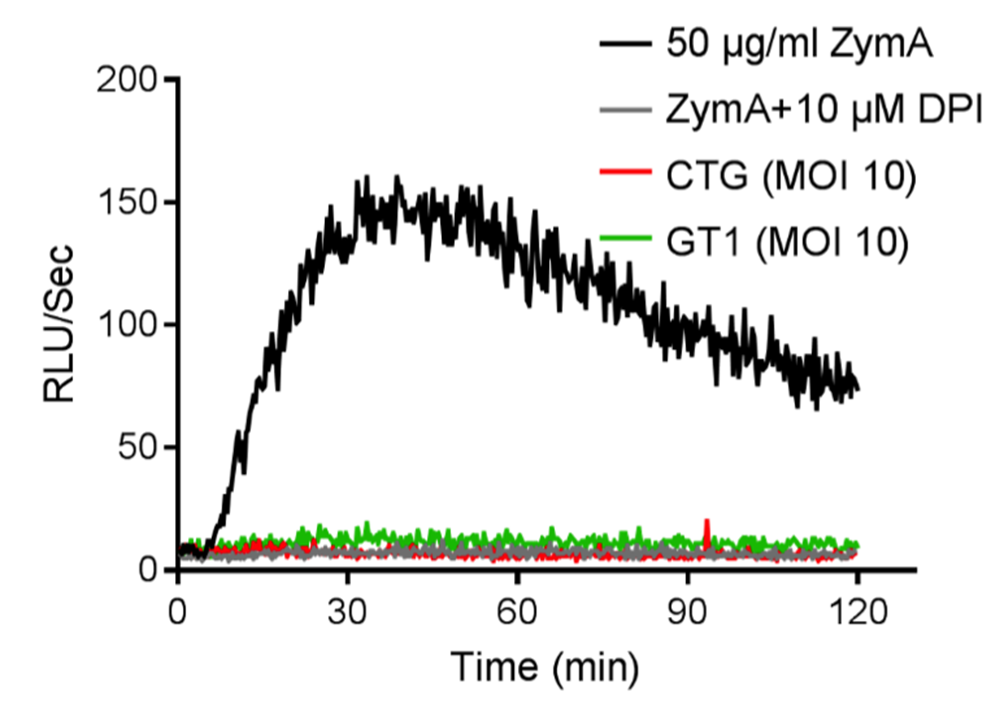

Supplement: FIG S2 [file mbo004184036sf2.tif]

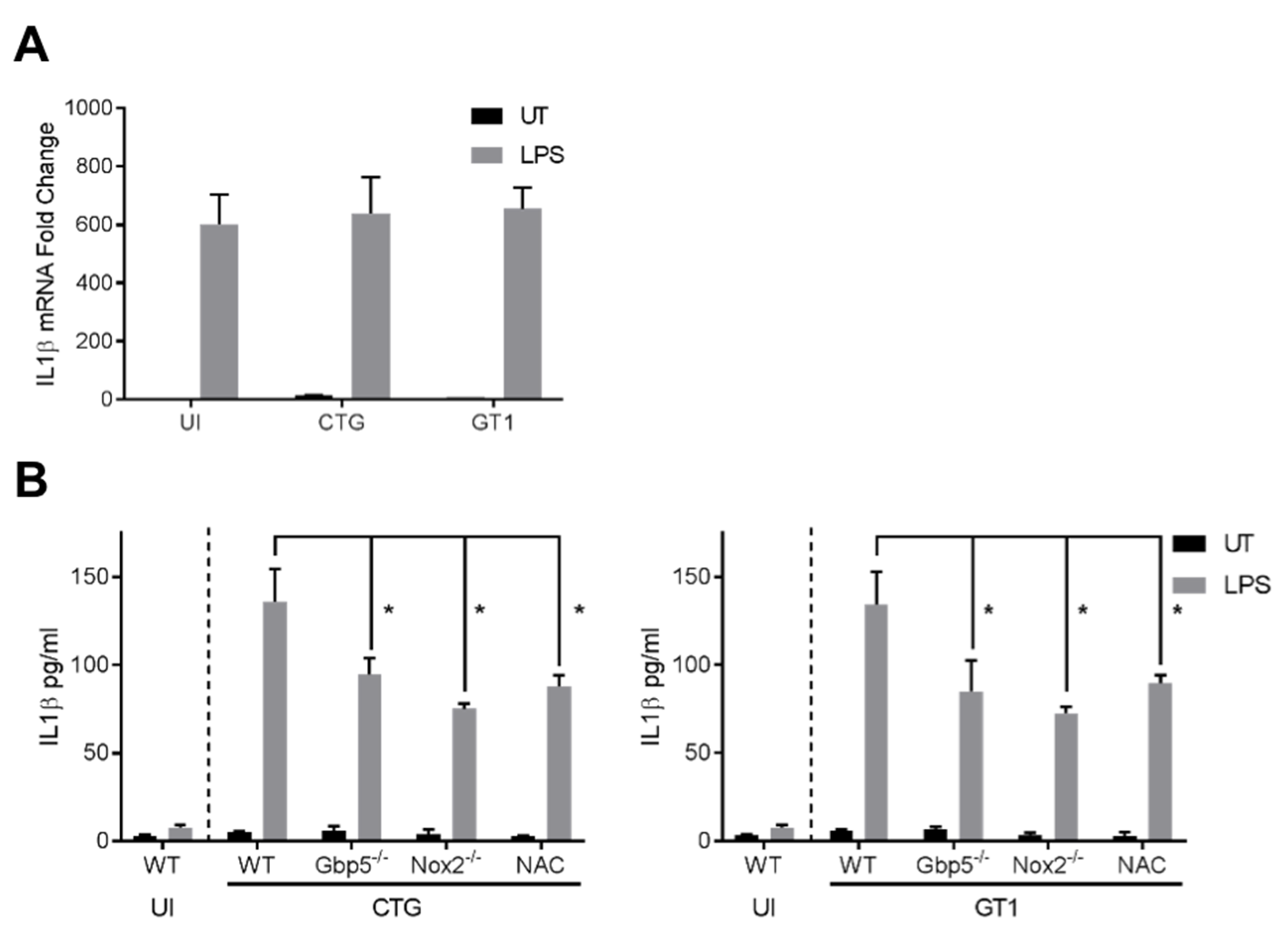

Supplement: FIG S3 [file mbo004184036sf3.tif]

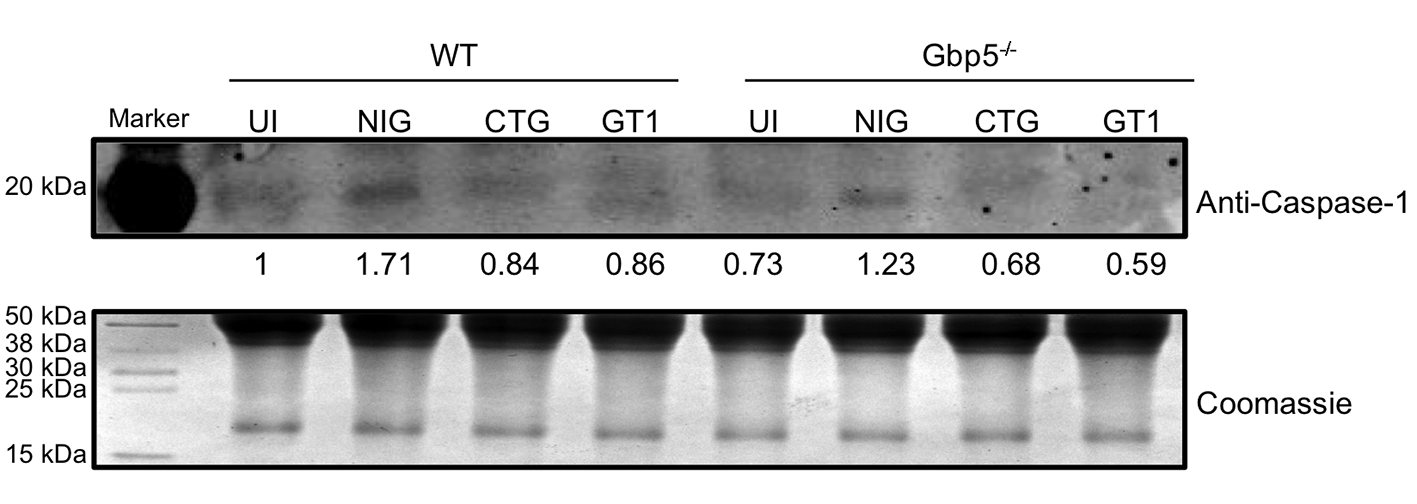

Supplement: FIG S4 [file mbo004184036sf4.tif]

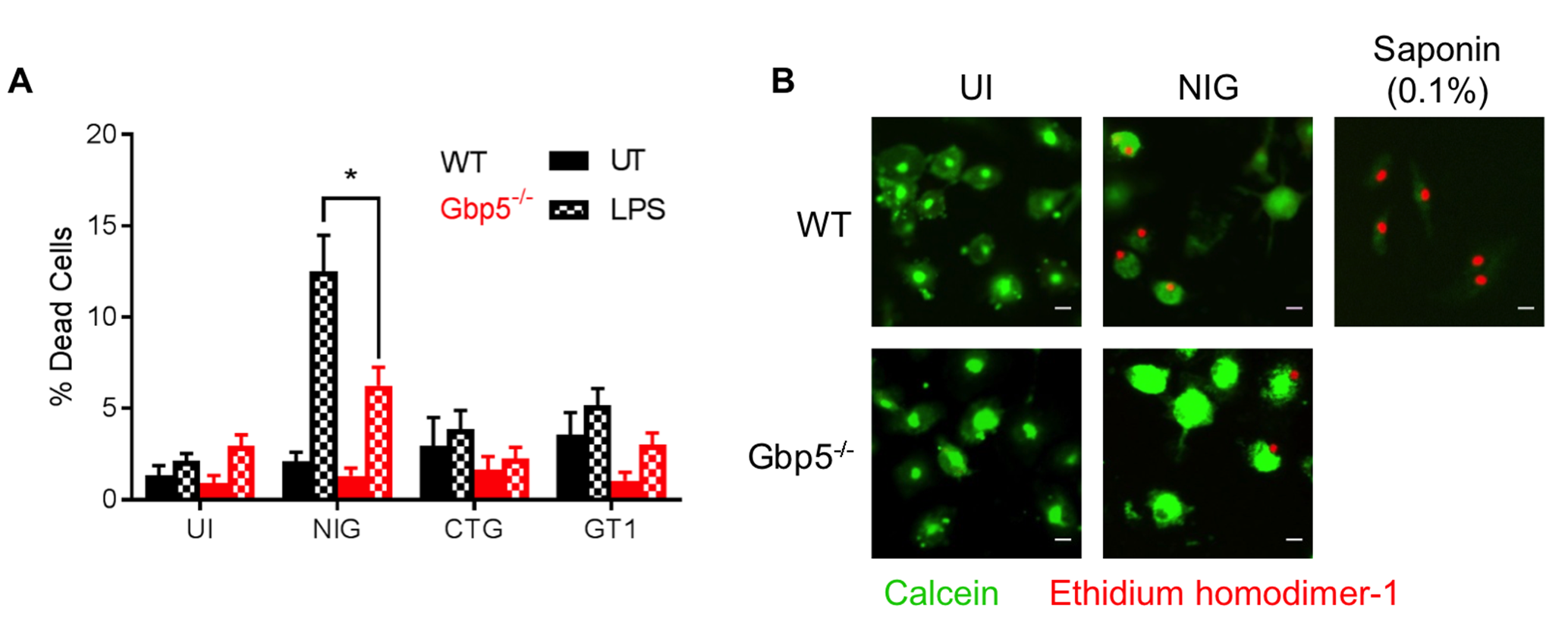

Supplement: FIG S5 [file mbo004184036sf5.tif]
