# Supplementary material for: NADPH Oxidase and Guanylate Binding Protein 5 Restrict Survival of Avirulent Type III Strains of Toxoplasma gondii in Naive Macrophages
Source: mBio. 2018 Aug 28;9(4):e01393-18. doi: 10.1128/mBio.01393-18 (PMC6113620; doi:10.1128/mBio.01393-18)
Supplement: TABLE S1 [file mbo004184036st1.docx]

**Table S1** Real time PCR primers

| **Gene** | **Forward (5' → 3')** | **Reverse (5' → 3')** |
| --- | --- | --- |
| Gbp1 | CGAGAAGCCAGAACATACCC | TGGTTGATGGTTCCTATGCTG |
| Gbp2 | TGAAGATGTTGAGAAGGGTGAC | GATCAGTTAGCTCCGTCACATAG |
| Gbp5 | GCAGGAGTTCTATCATAAACCAGG | CTCACTGTTTCTTTGGCGTTC |
| Gbp7 | TCCTGTGTGCCTAGTGGAAAA | CAAGCGGTTCATCAAGTAGGA |
| Irga6 | GGGTACTTACTTCCTAAAAATAGTTTTCT | TCACAGGACTTCAGCTTAATTAGA |
| IL-1β | ACGGACCCCAAAAGATGAAG | TTCTCCACAGCCACAATGAG |
| Actin | ACCTTCTACAATGAGCTGCG | CTGGATGGCTACGTACATGG |
